# Disseminated Pulmonary and Hepatic Cystic Echinococcosis Presenting as Suspected Pulmonary Embolism and Mimicking Metastatic Malignancy

**DOI:** 10.7759/cureus.115184

**Published:** 2026-08-25

**Authors:** Samarth E Joseph, Khansa S Makawi, Prakash Joseph, Hissa N Alsuwaidi

**Affiliations:** 1 Internal Medicine, Weill Cornell Medical College - Qatar, Doha, QAT; 2 General Internal Medicine, Hamad Medical Corporation, Doha, QAT

**Keywords:** albendazole, echinococcosis, echinococcus granulosus, neoplasm metastasis, positron emission tomography computed tomography, pulmonary embolism

## Abstract

Cystic echinococcosis (CE) is a chronic zoonotic infection caused by *Echinococcus granulosus* that most commonly affects the liver and lungs. In disseminated disease, the combination of bilateral pulmonary nodules, cavitary consolidation, mediastinal lymphadenopathy, and hepatic lesions can closely mimic metastatic malignancy on cross-sectional imaging and 18F-fluorodeoxyglucose positron emission tomography/computed tomography (FDG PET/CT). This case highlights disseminated CE mimicking pulmonary embolism (PE) and metastatic malignancy.

This is a case of a previously healthy 27-year-old woman who presented with acute dyspnea, pleuritic chest pain, fever, and productive cough and was referred for suspected PE; however, CT pulmonary angiography ruled out PE and revealed bilateral pulmonary nodules, cavitary right middle lobe consolidation, mediastinal lymphadenopathy, and multiple hepatic lesions initially interpreted as metastatic malignancy. FDG PET/CT showed intensely hypermetabolic pulmonary and mediastinal disease (SUVmax (maximum standardized uptake value), 15.6), while the hepatic lesions were isometabolic, suggesting a separate etiology. Liver MRI showed well-defined cystic lesions across multiple hepatic segments consistent with hydatid cysts, and *Echinococcus *serology was positive (titer, 1:640). Bronchoalveolar lavage (BAL) was negative for malignancy and alternative infections. She was treated with albendazole 400 mg twice daily, and five-week follow-up CT showed cavitary transformation of pulmonary nodules consistent with treatment-related cyst degeneration. Disseminated CE can mimic both PE and metastatic malignancy. In patients from endemic regions, discordant FDG PRT/CT findings - hypermetabolic pulmonary lesions with isometabolic hepatic cysts - should prompt consideration of CE. A multidisciplinary approach integrating multimodal imaging and serology is essential for accurate diagnosis and to avoid unnecessary invasive procedures.

## Introduction

Cystic echinococcosis (CE) is a chronic zoonotic infection caused by the larval stage of the tapeworm *Echinococcus granulosus* sensu lato. It is estimated that over one million people are infected worldwide at any given time, accounting for more than one million disability-adjusted life years lost annually [1]. CE is most prevalent in pastoral communities across the Mediterranean basin, Central Asia, South America, and East Africa, where the life cycle of the organism is maintained between domestic dogs and livestock [1]. Humans serve as accidental intermediate hosts following ingestion of contaminated food, water, or soil [2]. The liver and lungs are the most commonly affected organs, and cysts may grow slowly over years before becoming clinically apparent [1,2]. 

The diagnosis of CE can be challenging, particularly when cysts present with atypical imaging features. Due to their varying appearances at different developmental stages, hydatid cysts can mimic a range of pathologies from simple cysts to malignancy [3]. In nonendemic settings, metastatic tumor is a major diagnostic consideration when multiple hepatic and pulmonary lesions are identified [4]. Furthermore, complicated or infected cysts may demonstrate intense 18F-fluorodeoxyglucose (FDG) uptake on positron emission tomography/computed tomography (PET/CT), further compounding the resemblance to malignant disease [5]. Conversely, the acute presentation of pulmonary CE with dyspnea, pleuritic chest pain, and pleural effusion may initially raise suspicion for pulmonary embolism (PE) [6].

Here, we present a case of disseminated pulmonary and hepatic CE in a young Algerian woman whose initial clinical presentation mimicked acute PE and the subsequent radiologic findings closely resembled metastatic malignancy. This case highlights the diagnostic pitfalls of CE and the importance of maintaining a broad differential diagnosis in patients from endemic regions presenting with multiorgan cystic disease.

## Case presentation

A 27-year-old Algerian woman with morbid obesity (BMI, 43 kg/m²) and no known past medical or surgical history presented with the acute onset of pleuritic chest pain and shortness of breath while traveling with her children. The symptoms started suddenly and were not preceded by trauma, recent illness, or any apparent precipitating event.

Later the same evening, she developed a low-grade fever accompanied by a non-productive cough. She attended a private clinic, where PE was suspected based on her clinical presentation. She received therapeutic-dose enoxaparin and was referred to our hospital for further evaluation and management. The patient denied hemoptysis, weight loss, night sweats, anorexia, recent tuberculosis exposure, smoking, or alcohol use. She had last traveled to Algeria approximately six months before the presentation. She denied known contact with livestock, sheep, dogs, or other animals and had no known family history of hydatid disease.

On arrival, her chest pain and dyspnea had improved compared with her initial presentation; her vital signs were stable, with a temperature of 37.1°C, heart rate of 78 beats/min, blood pressure of 114/74 mmHg, respiratory rate of 16 breaths/min, and oxygen saturation of 99% on room air. Respiratory system examination revealed diminished breath sounds in the right lower lung zone and crackles in the right mid and lower lung zones without evidence of respiratory distress. The remainder of the systemic examination was unremarkable.

Initial laboratory investigations showed a white blood cell count of 5 × 10³/µL with 56.4% neutrophils and an eosinophil count of 0.40 × 10³/µL, hemoglobin of 12.8 g/dL, and platelet count of 167 × 10³/µL (Table 1). C-reactive protein was 88 mg/L. Renal function was normal with blood urea nitrogen of 3.2 mmol/L and creatinine of 44 µmol/L. Baseline liver profile showed an aspartate aminotransferase level of 12 U/L, alanine aminotransferase level of 13 U/L, alkaline phosphatase level of 76 U/L, total bilirubin level of 18 µmol/L, and albumin level of 35 g/L. Coagulation studies showed a prothrombin time of 13.5 seconds, international normalized ratio of 1.2, and activated partial thromboplastin time of 35.5 seconds. Hepatitis B and C serology, human immunodeficiency virus testing, antineutrophil cytoplasmic antibody testing, and a respiratory viral panel were negative. Two sets of blood cultures obtained on admission showed no growth. Electrocardiography demonstrated normal sinus rhythm without evidence of right heart strain.

**Table 1:** Laboratory investigations AFP: Alpha-fetoprotein; ALP: Alkaline phosphatase; ALT: Alanine aminotransferase; ANA: Antinuclear antibody; ANCA: Antineutrophil cytoplasmic antibody; aPTT: Activated partial thromboplastin time; AST: Aspartate aminotransferase; CA: Cancer antigen; CEA: Carcinoembryonic antigen; CRP: C-reactive protein; CTD: Connective tissue disease; HIV: Human immunodeficiency virus; IgA: Immunoglobulin A; IgM: Immunoglobulin M; INR: International normalized ratio; MTB: Mycobacterium tuberculosis; PCR: Polymerase chain reaction; PMNLs: Polymorphonuclear leukocytes; PT: Prothrombin time; TB: Tuberculosis; WBC: White blood cell.

Parameter	Patient value	Reference range
Initial laboratory investigations		
WBC	5 × 10³/µL	(Normal: 4.0–10 x 10³/µL)
Neutrophils	2.8 x 10³/µL	(Normal: 2.0–7.0 x 10³/µL)
Eosinophils	0.4 x 10³/µL	(Normal: 0.2–0.5 x 10³/µL)
Hbg	12.8 g/dL	(Normal: 12–15 g/dL)
Platelets	167 x 10³/µL	(Normal: 150–410 x 10³/µL)
CRP	88.0 mg/L	(Normal: 0.0–5.0 mg/L)
Urea	3.2 mmol/L	(Normal: 2.5–7.8 mmol/L)
Creatinine	44 µmol/L	(Normal: 44–80 µmol/L)
AST	12 U/L	(Normal: 0–32 U/L)
ALT	13 U/L	(Normal: 0–33 U/L)
ALP	76 U/L	(Normal: 35–104 U/L)
Bilirubin T	18 µmol/L	(Normal: 0–21 µmol/L)
Albumin	35 g/L	(Normal: 35–50 gm/L)
PT	13.5 s	(Normal: 9.4–12.5 s)
INR	1.2	(Critical high >4.9)
aPTT	35.4 s	(Normal: 25.1–36.5 s)
Viral panel		
Hepatitis A IgM	Negative	
Hepatitis B surface antigen	Negative	
Hepatitis B antibody	<2	
Hepatitis C	Negative	
HIV Ag/Ab	Negative	
Blood glucose	4.9 mmol/L	
ANCA	Negative	
ANA CTD interpretation	Negative	
Aspergillus galactomannan antigen	Negative	
AFP	2 IU/mL	(Normal: 0–6 IU/mL)
Ova and parasites in stool	Negative	
Fungal culture		
Preliminary report	No fungus isolated	
Macroscopic examination report	Hazy, blood-stained	
Tuberculosis		
IgA	1.41 g/L	(Normal: 0.7–4 gm/L)
QuantiFERON-TB Gold Plus	Negative	
TB PCR	Negative	
TB PCR for MTB	Not detected	
Acid-fast bacilli smear	No acid-fast bacilli	
Acid-fast bacilli culture	No growth at 42 days	
Tumor markers		
CA 125	4.7	(Normal: 0–35 U/mL)
CA 15-3	3.3	(Normal: 0–34.5 U/mL)
CA 19-9	2.7	(Normal: 0–27 U/mL)
CEA	0.5 µg/L	
Bronchoalveolar lavage		
Lower respiratory tract culture	Normal upper respiratory flora isolated	
Gram stain	Profuse polymorphonuclear leukocytes	
	Scanty epithelial cells	
	No organisms seen	
Cytology report	Microscopic description	
	Reactive alveolar macrophages	
	Epithelial cells in a background of PMNLs	
Parasitic serology		
*Echinococcus *antibody	Positive	
*Echinococcus *titer	Positive, 1:640	
Treponema pallidum	Nonreactive	

**Figure 1:**
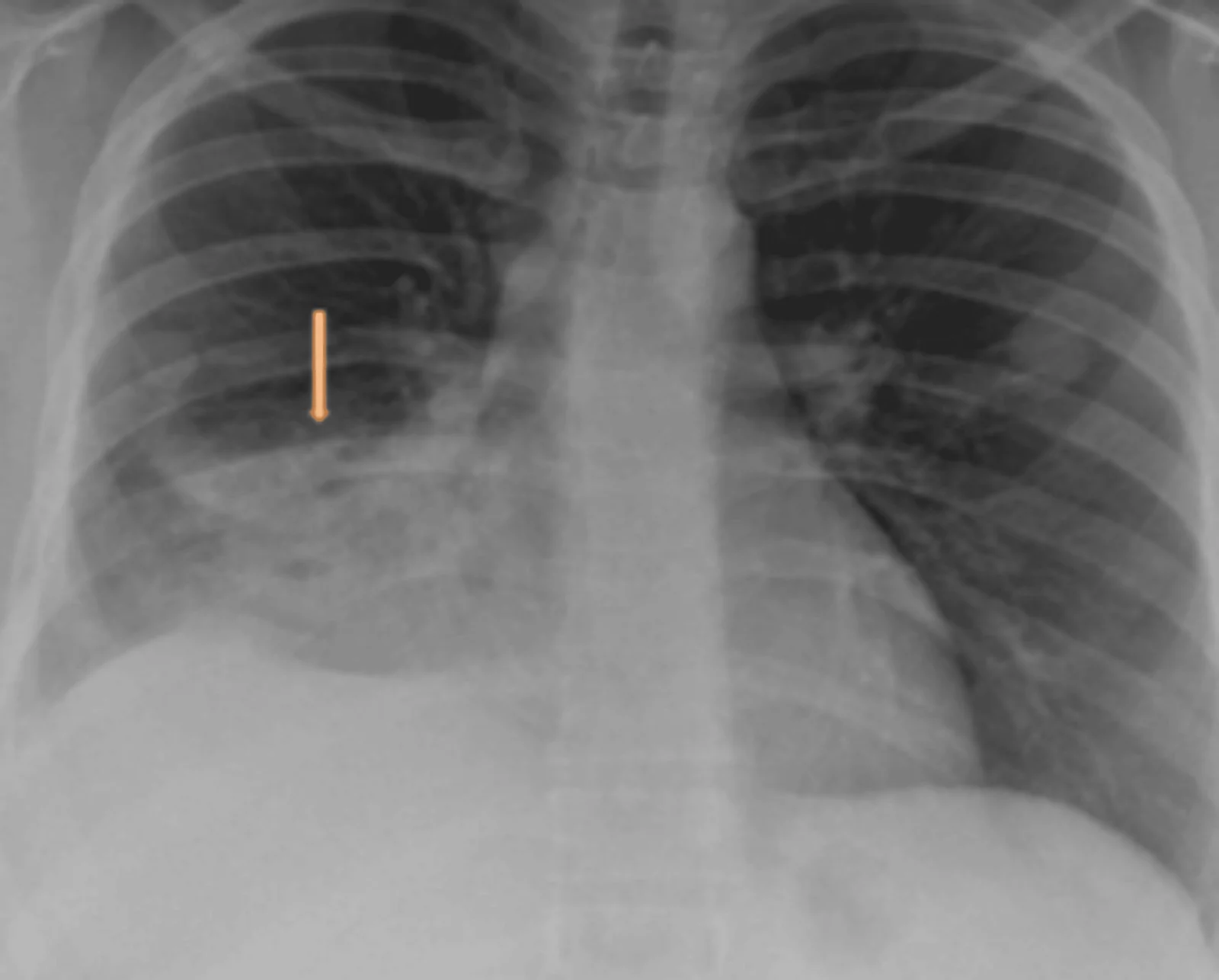
Chest X-ray (on admission) showing the air space opacity concerning for infection The arrow points toward the airspace opacity silhouetting the right heart border in the lower lung zone.

A chest X-ray revealed airspace opacity silhouetting the right heart border in the lower lung zone (Figure 1), and a computed tomography pulmonary angiography (CTPA) performed to evaluate for PE revealed no filling defects within the pulmonary arterial vasculature, effectively excluding the diagnosis of acute PE.

**Figure 2:**
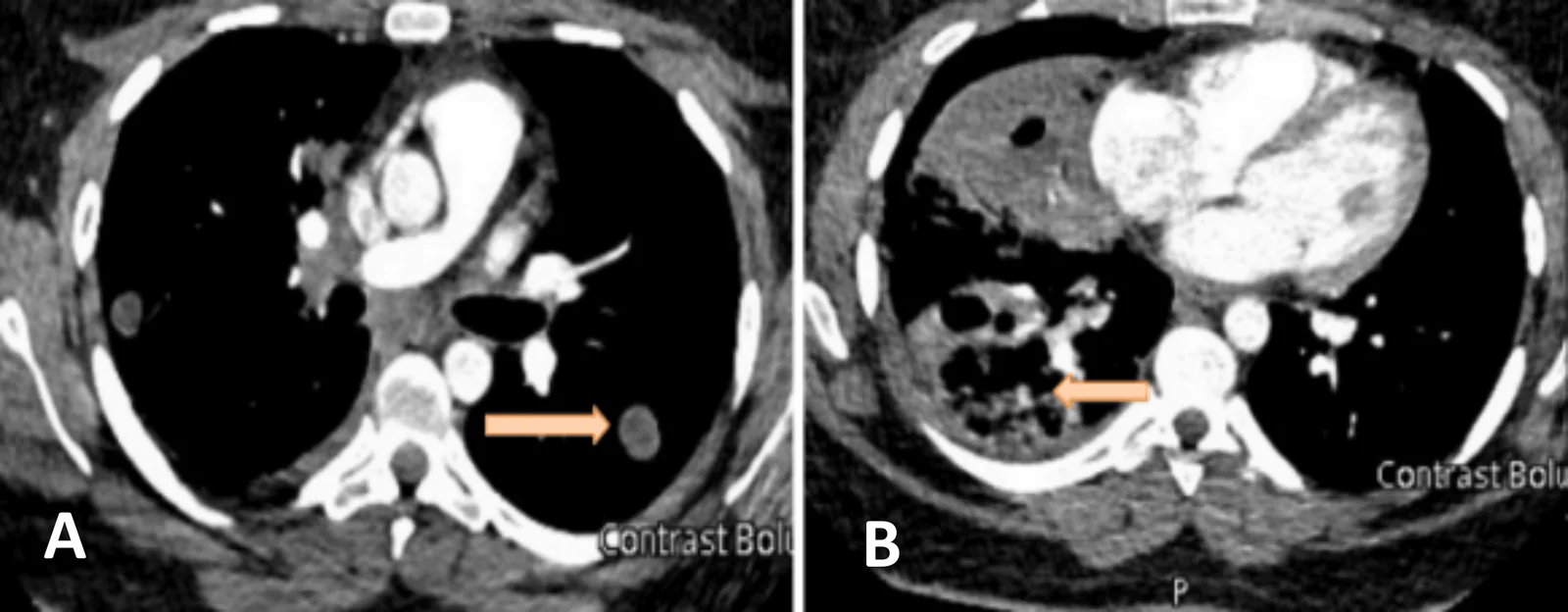
CT scan of the chest on admission: (A) cross-section of the upper lung field and (B) cross-section of the mid-lung field (A) Nodular opacity in the upper left lung field representing an *Echinococcus *cyst. (B) Consolidation with necrosis and cavitary liquefactive changes in the right mid-lung field.

Multiple bilateral nodular opacities were identified throughout the lung fields, the largest measuring 1.8 × 1.7 cm abutting the mediastinal pleura of the right middle lobe (Figure 2, Panel A). Collapse and consolidation of the right middle lobe with cavity formation were noted (Figure 2, Panel B) along with consolidation involving the right lower lobe with areas of liquefaction. A minimal right-sided pleural effusion was present. No pneumothorax or pericardial effusions were identified. Multiple minimally enlarged mediastinal lymph nodes were observed, the largest measuring 1.5 × 1.0 cm in the right paratracheal region.

**Figure 3:**
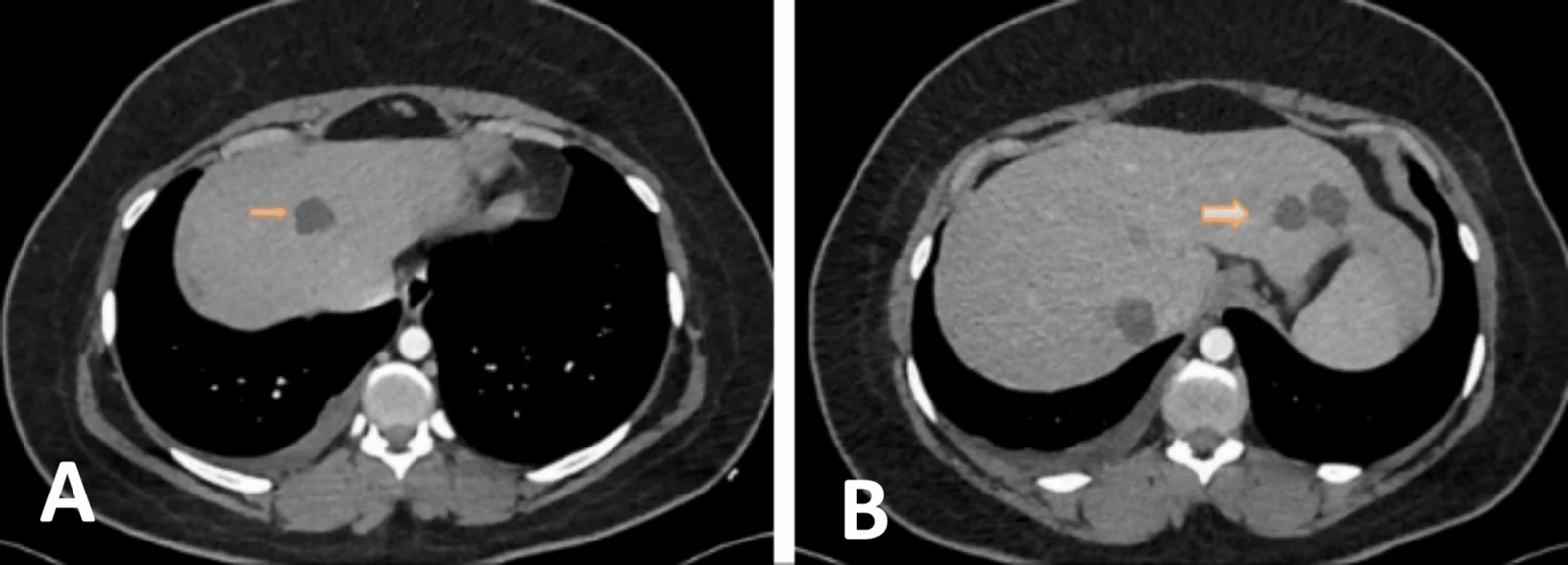
CT scan of the liver on admission: (A, B) cross-sectional images of the liver showing hypodense lesions The arrows indicate hypodense hepatic lesions representing cystic involvement by *Echinococcus*.

Upper abdominal images (Figure 3, Panels A and B) revealed multiple hypodense hepatic lesions. The radiologic conclusion favored neoplastic pathology with metastasis as the most likely unifying diagnosis. Given the concurrent fever and productive cough, empiric antimicrobial therapy with intravenous ampicillin-sulbactam and oral azithromycin was initiated to address a possible secondary bacterial infection.

**Figure 4:**
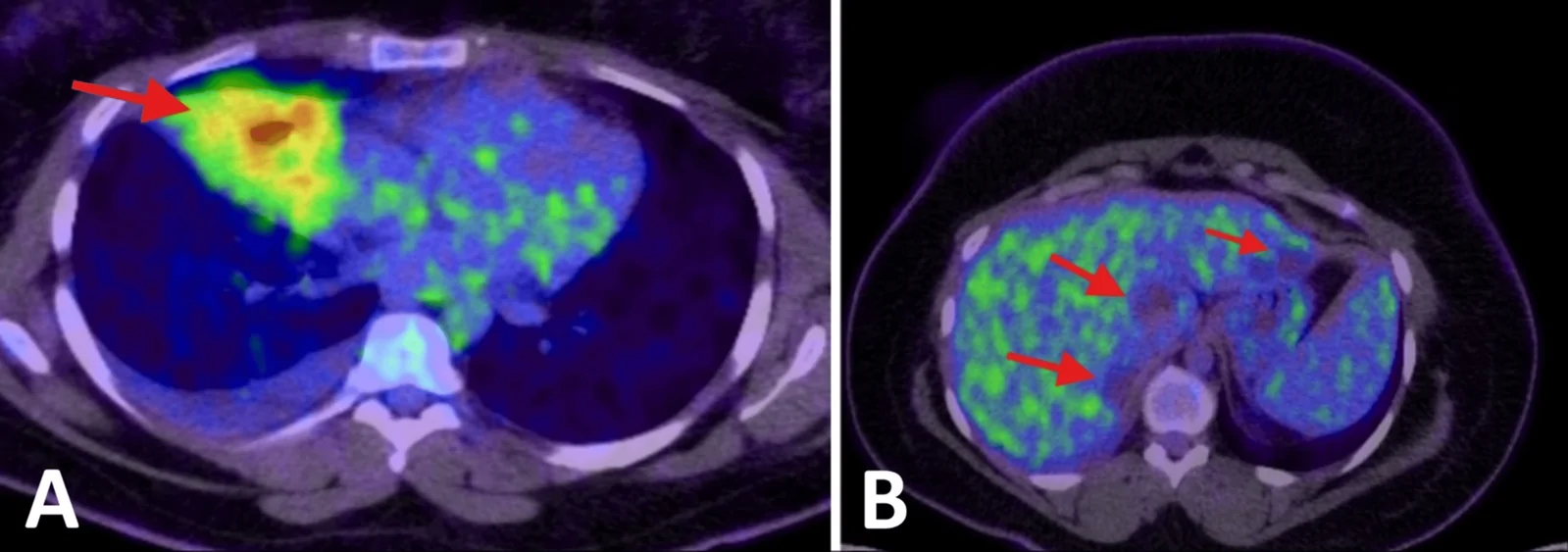
Full-body PET scan during admission: (A) Cross-sectional image of the mid-lung field and (B) cross-sectional image of the upper liver (A) Intense hypermetabolic uptake in the right middle lobe, with consolidation and a central air-containing component suspicious for necrotic change. (B) Low-attenuation bilobar hepatic nodules that are isometabolic relative to the surrounding liver parenchyma.

Given the concern for malignancy, 18F-FDG PET/CT was performed for further characterization. The study demonstrated intense hypermetabolic uptake in the right middle lobe consolidation (Figure 4, Panel A), with a central air-containing component suspicious for necrotic change. Multiple foci of intense uptake (SUVmax (maximum standardized uptake value), 15.6) were identified in the right upper paratracheal, right lower paratracheal, and subcarinal lymph nodes, raising suspicion for metastatic disease. A 1.6-cm right upper lobe subpleural nodule demonstrated mildly increased uptake, whereas a 1.9-cm left upper lobe nodule abutting the fissure was non-FDG-avid. No abnormal uptake was identified in the head and neck, axillary lymph nodes, breasts, or osseous structures. Notably, the low-attenuation bilobar hepatic nodules (Figure 4, Panel B) were isometabolic relative to the surrounding liver parenchyma (background liver SUVmax, 4.9). The nuclear medicine interpretation concluded that these lesions likely represented a separate etiology unrelated to the pulmonary and mediastinal disease and recommended liver MRI for further characterization.

**Figure 5:**
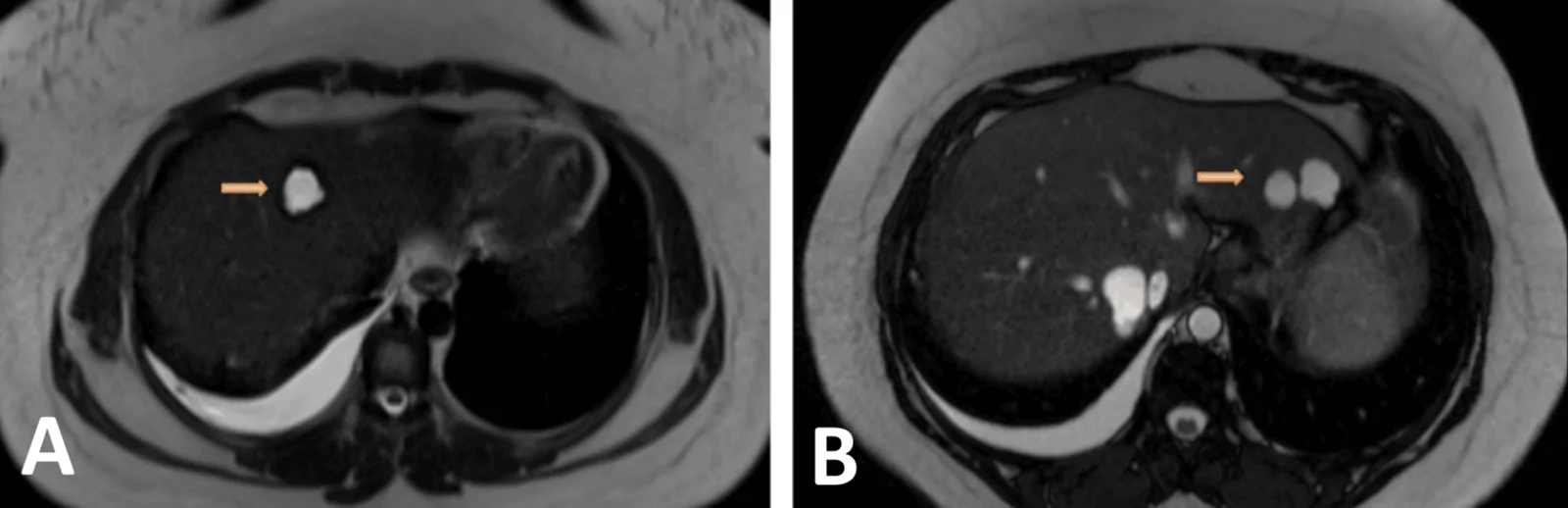
MRI scan of the liver on admission: (A, B) Cross-sectional images of the upper part of the liver showing multiple round, hyperintense nodules in the right and left lobes of the liver, with an associated pleural effusion The arrows indicate round nodules representing cystic changes in the liver.

Magnetic resonance imaging (MRI) of the liver and abdomen with contrast was performed for further characterization of the hepatic lesions. The study confirmed the right middle lobe consolidation and partial collapse, with multiple cystic lesions, some of which appeared collapsed, and a right pleural effusion. Within the liver (Figure 5, Panels A and B), numerous well-defined cystic lesions measuring up to 25 mm in maximum dimension were identified across segments I, II, VI, VII, and VIII. No abnormalities were identified in the genitourinary, adrenal, gastrointestinal, vascular, nodal, or peritoneal compartments. The thoracoabdominal wall was unremarkable. The radiologic findings were suggestive of hepatic and pulmonary hydatid cysts, representing a pivotal shift in the diagnostic trajectory from suspected malignancy to parasitic disease. *Echinococcus *serology was subsequently performed and returned positive, with a titer of 1:640.

Given the atypical pulmonary findings and ongoing concern for infection or malignancy, flexible bronchoscopy was performed. Examination of the airways revealed normal nasopharyngeal mucosa, mobile vocal cords, a normal trachea with a sharp carina, and no endobronchial lesions or active bleeding in either bronchial tree. Bronchoalveolar lavage (BAL) was obtained from the right middle and lower lobes, and samples were submitted for Gram stain and bacterial culture, mycobacterial culture, fungal culture, and ova and parasite evaluation, which were negative. The combination of positive serology, compatible MRI findings, pulmonary and hepatic cystic disease, and negative alternative investigations supported the diagnosis of disseminated CE (Table 2).

**Table 2:** Timeline of patient evaluation and diagnosis: pulmonary and hepatic echinococcosis Diagnostic pathway: Presentation (January 18, 2026) → CTA abnormality (January 19, 2026) → PET scan, CT abdomen, and MRI of the liver (January 21, 2026) → *Echinococcus* antibody titer sent (January 21, 2026) → serology reported and bronchoscopy/BAL performed (January 22, 2026) → further diagnostic assessment and treatment → follow-up CT of the thorax (February 25, 2026). AFB: Acid-fast bacilli; BAL: Bronchoalveolar lavage; CTA: Computed tomography angiography; CT: Computed tomography; MRI: Magnetic resonance imaging; PCR: Polymerase chain reaction; PET: Positron emission tomography; TB: Tuberculosis.

Date	Clinical Event/Investigation	Result/Key Finding
January 18, 2026	Patient arrival/admission	Initial clinical assessment. CBC and serum electrolytes were performed.
January 19, 2026	Respiratory viral panel	Negative.
January 19, 2026	Sputum AFB smear and TB PCR	Negative.
January 19, 2026	Sputum studies	Samples collected; final results received on January 28, 2026.
January 19, 2026	CT angiography (CTA)	First significant radiological findings were identified, prompting further diagnostic evaluation.
January 20, 2026	Bronchial aspiration studies	Reports received.
January 21, 2026	Whole-body PET scan	Performed to further characterize lesions and assess the extent of disease.
January 21, 2026	CT abdomen	Performed to assess abdominal/hepatic involvement.
January 21, 2026	MRI abdomen and liver	Performed for detailed characterization of hepatic lesions.
January 21, 2026	*Echinococcus *antibody titer	A blood sample was collected and sent for *Echinococcus *serology.
January 22, 2026	*Echinococcus *antibody titer	Result received/reported.
January 22, 2026	Bronchoscopy with BAL	Performed for further evaluation of pulmonary lesions.
January 22, 2026	BAL infectious workup	TB studies, AFB smear/PCR, *Aspergillus *studies, and fungal cultures were negative.
January 28, 2026	Sputum studies	Final reports from samples collected on January 19, 2026 were received.
February 25, 2026	Follow-up CT thorax after treatment	Performed to assess radiological response to treatment.

A multidisciplinary discussion between the pulmonology, infectious disease, and interventional radiology teams was undertaken, and the patient was initiated on albendazole 400 mg orally twice daily for six months. A planned interventional radiology-guided percutaneous liver biopsy was subsequently cancelled after the evolving clinical picture favored hydatid disease, given the recognized risks of cyst rupture, anaphylaxis, and peritoneal dissemination. The final clinical impression was disseminated CE with pulmonary and hepatic involvement complicated by suspected secondary bacterial infection of a pulmonary hydatid cyst rather than metastatic malignancy. The patient's symptoms improved with antimicrobial therapy and initiation of anthelmintic treatment. She was discharged in stable condition on albendazole, with instructions for outpatient monitoring of hepatic transaminases and complete blood counts every two weeks during therapy.

**Figure 6:**
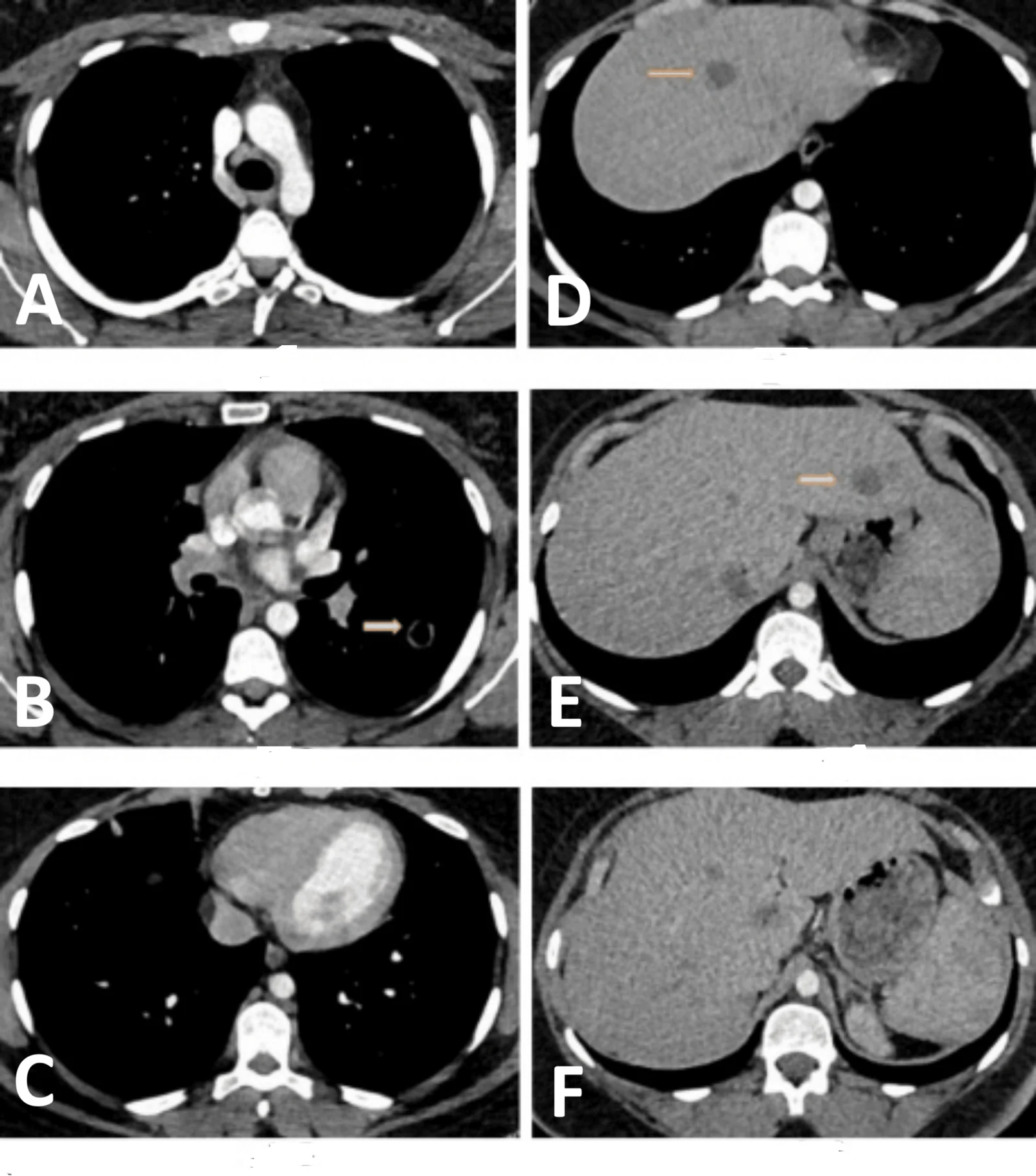
CT scan of the chest and abdomen after five weeks of treatment: (A-C) cross-sectional images of the upper and mid-lung fields; (D-F) cross-sectional images of the upper right and left liver lobes A repeat scan showed resolution of the pulmonary cavitary and liquefactive changes, with stable hepatic *Echinococcus *cysts. (B) The arrow indicates cavitary transformation consistent with treatment-related cyst degeneration. (D, E) The arrows indicate multiple hepatic hypodense lesions that remained stable.

Follow-up imaging approximately five weeks after treatment initiation with repeat computed tomography of the chest (Figure 6) showed findings consistent with treatment response. Several previously solid pulmonary nodules in the right upper lobe, right middle lobe, and left lower lobe had undergone cavitary transformation (Figure 6, Panel B), consistent with treatment-related cyst degeneration. The previously noted ​​​​​right middle lobe consolidation had resolved (Figure 6, Panel C). The right pleural effusion had also resolved, and mediastinal lymph nodes had decreased in size. Multiple hepatic hypodense lesions remained stable (Figure 6, Panels D and E). Overall, these findings were interpreted as interval cavitary transformation of pulmonary hydatid cysts, consistent with an early therapeutic response to albendazole. Longer follow-up is required to assess complete resolution of the imaging findings, which may be a limitation of this case.

## Discussion

This case illustrates two clinically important diagnostic pitfalls in a young woman from an endemic region. First, disseminated pulmonary and hepatic CE presented acutely with symptoms that prompted evaluation for PE. Second, after PE was excluded, the combination of bilateral pulmonary nodules, cavitary consolidation, mediastinal lymphadenopathy, and multiple hepatic lesions closely mimicked metastatic malignancy on both cross-sectional imaging and FDG PET/CT. Recognition of CE required integration of multimodal imaging, serologic testing, and bronchoscopic evaluation, ultimately averting unnecessary invasive procedures and enabling appropriate medical therapy.

CE is a chronic zoonotic infection caused by the larval stage of *Echinococcus granulosus* sensu lato. The liver (~70%-80%) and lungs (~15%-20%) are the most commonly affected organs [1,4]. Algeria is recognized as a hyperendemic country within the Mediterranean basin, with the sheep/dog strain (G1) predominating in both human and animal hosts [7,8]. The incubation period is prolonged and often spans years, with up to 60% of cases remaining asymptomatic [3]. In North Africa, most symptomatic cases occur in adults aged 21-40 years [3]. Notably, many patients do not recall a specific animal exposure, as transmission may occur through environmental contamination with *Echinococcus *eggs in soil, water, or food without direct livestock contact [2,9]. The present patient denied known animal exposure, consistent with this epidemiologic pattern.

The radiologic presentation in this case was highly suggestive of disseminated malignancy. CTPA demonstrated bilateral pulmonary nodules, right middle lobe consolidation with cavitation, mediastinal lymphadenopathy, and multiple hepatic lesions. This constellation was interpreted by the reporting radiologist as most likely representing neoplastic pathology with metastasis. Hydatid disease can have varying imaging appearances depending on the developmental stage of the cysts, and complicated or atypical cysts may be indistinguishable from solid neoplasms on CT [2,3]. MRI was instrumental in redirecting the diagnostic trajectory by demonstrating well-defined cystic characteristics of the hepatic lesions across multiple segments, features more consistent with hydatid cysts than solid metastatic deposits [10]. This case reinforces the value of MRI for differentiating CE from neoplastic disease when CT findings are equivocal.

FDG PET/CT further compounded the initial suspicion for malignancy, demonstrating intensely hypermetabolic uptake (SUVmax, 15.6) in the right middle lobe consolidation and mediastinal lymph nodes. Infectious and inflammatory processes, including complicated hydatid cysts, are well-recognized causes of false-positive FDG uptake [5,11]. Infected or ruptured *Echinococcus *cysts generate a robust perilesional inflammatory response with neutrophilic and macrophage infiltration, accounting for intense FDG avidity [12]. Critically, the hepatic lesions in this case were isometabolic relative to the surrounding liver parenchyma, which is a finding consistent with prior data. This shows that uncomplicated CE liver cysts are characteristically FDG-negative, in contrast to the hypermetabolic pattern seen in alveolar *Echinococcus *or malignancy [13]. This discordance between intensely hypermetabolic pulmonary lesions and isometabolic hepatic cysts should prompt consideration of an infectious etiology, particularly CE, rather than a single unifying malignant process. A prior case report similarly described pulmonary echinococcosis mimicking lung metastases, emphasizing the high negative predictive value of FDG PET for characterizing indeterminate pulmonary nodules greater than 1 cm [8].

*Echinococcus *serology confirmed the diagnosis with a positive titer of 1:640. Serologic sensitivity for CE varies from 60% to 90% depending on the assay, cyst stage, and organ involvement, with ELISA (enzyme-linked immunosorbent assay) demonstrating the highest sensitivity among available assays [14]. Sensitivity is notably lower for isolated pulmonary cysts compared with hepatic cysts, making the concurrent hepatic involvement in this case advantageous for serologic confirmation [15]. BAL and microbiologic workup were negative for mycobacterial, fungal, and bacterial pathogens, further narrowing the differential. A notable laboratory finding was the absence of peripheral eosinophilia, which may have contributed to the initial low index of suspicion for parasitic disease. Intact echinococcal cysts are antigenically sequestered within host tissue and are not typically associated with peripheral blood eosinophilia unless there is disruption of sequestration or cyst rupture [16]. In a large retrospective series, only 24.8% of patients with CE demonstrated eosinophilia, and its presence was associated with complications such as cyst rupture or fistula formation [16]. The normal eosinophil count in this case is therefore consistent with the expected immunologic profile of CE and should not be used to exclude the diagnosis.

Medical therapy with albendazole 400 mg twice daily was initiated as the primary treatment, given the disseminated nature of the disease, with multiple pulmonary and hepatic cysts rendering surgical resection impractical. Albendazole interferes with parasite glucose metabolism and β-tubulin structure and is considered parasitostatic rather than parasiticidal [1]. The American College of Gastroenterology (ACG) Clinical Guideline on Focal Liver Lesions recommends medical therapy for inoperable cases with multiple cysts and notes that continuous dosing regimens are now preferred over previously recommended cyclical regimens [2]. However, medical monotherapy has important limitations, as a large systematic review demonstrated that more than 40% of hydatid cysts remain active or reactivate after two years of medical monotherapy [2]. The planned percutaneous liver biopsy was appropriately cancelled given the recognized risks of cyst rupture, anaphylaxis, and peritoneal dissemination associated with percutaneous puncture of echinococcal cysts [2,17]. Follow-up CT at five weeks demonstrated cavitary transformation of previously solid pulmonary nodules, resolution of the right middle lobe consolidation, and decreased mediastinal lymphadenopathy, consistent with treatment-related cyst degeneration and supporting the diagnosis ex juvantibus.

## Conclusions

This case demonstrates that disseminated CE can present acutely with symptoms mimicking PE and produce imaging findings on CT and FDG PET/CT that closely resemble metastatic malignancy. The discordance between intensely hypermetabolic pulmonary lesions and isometabolic hepatic cysts on PET/CT was a critical diagnostic clue that suggested an infectious rather than neoplastic etiology. MRI and serologic testing were pivotal in establishing the correct diagnosis and redirecting management away from invasive biopsy toward appropriate medical therapy with albendazole. The absence of peripheral eosinophilia and lack of reported animal exposure should not be used to exclude the diagnosis. CE should remain in the differential diagnosis of multiorgan cystic disease in patients from endemic regions, and a multidisciplinary approach integrating multimodal imaging, serology, and endoscopic evaluation is essential for accurate diagnosis and to avoid unnecessary invasive procedures.
